# A ketogenic diet improves vascular hyperpermeability in type 2 diabetic mice by downregulating vascular pescadillo1 expression

**DOI:** 10.1111/jcmm.17744

**Published:** 2023-04-15

**Authors:** Song Wang, Jielin Zhou, Jing Lu, Yan Lin, Shuaishuai Liu, Keyang Chen

**Affiliations:** ^1^ Department of Ophthalmology, The Second Affiliated Hospital Anhui Medical University Hefei Anhui China; ^2^ Department of Nutrition and Food Hygiene, School of Public Health Anhui Medical University Hefei Anhui China; ^3^ Department of Oncology, Anhui Provincial Cancer Hospital The First Affiliated Hospital of the University of Science and Technology of China Hefei Anhui China; ^4^ Department of Health Inspection and Quarantine, School of Public Health Anhui Medical University Hefei Anhui China; ^5^ Department of Diabetic Retinopathy AIER Hefei Eye Hospital Affiliated to Anhui Medical University Hefei Anhui China

**Keywords:** diabetes, KD, PES1, vascular permeability, β‐HB

## Abstract

The role of pescadillo1 (PES1) in regulating vascular permeability has been unknown. This study probes the role of PES1 and its mediated molecular mechanism in modulating vascular hyperpermeability in diabetic mice. Male C57BL/6J and *db/db* mice were fed a standard diet and a ketogenic diet (KD). Meanwhile, mouse vascular endothelial cells (MVECs) were treated with β‐hydroxybutyric acid (β‐HB), *Pes1* siRNA or a *Pes1* overexpression plasmid. Additionally, knockout (KO) of *Pes1* in mice was applied. After 12 weeks of feedings, enhanced vascular PES1 expression in diabetic mice was inhibited by the KD. The suppression of PES1 was also observed in β‐HB‐treated MVECs. In mice with *Pes1* KO, the levels of vascular VEGF and PES1 were attenuated, while the levels of vascular VE‐cadherin, Ang‐1 and Occludin were upregulated. Similar outcomes also occurred after the knockdown of *Pes1* in cultured MVECs, which were opposite to the effects induced by PES1 overexpression in MVECs. In vitro and in vivo experiments showed that high glucose concentration‐induced increases in vascular paracellular permeability declined after MVECs were treated by β‐HB or by knockdown of *Pes1*. In contrast, increases in vascular permeability were induced by overexpression of *Pes1*, which were suppressed by coadministration of β‐HB in cultured endothelial cells. Similarly declines in vascular permeability were found by *Pes1* knockdown in diabetic mice. Mechanistically, β‐HB decreased PES1‐facilitated ubiquitination of VE‐cadherin. The KD suppressed the diabetes‐induced increase in PES1, which may result in vascular hyperpermeability through ubiquitination of VE‐cadherin in type 2 diabetic mice.

## 1 INTRODUCTION

Type 2 diabetes mellitus (T2DM) accounts for approximately 90% of cases of diabetes.^1^, ^2^ Genetic, environmental and metabolic risk factors are interrelated and contribute to the development of T2DM.^3^ Many diabetic patients suffer from a lot of secondary complications manifested as retinal microangiopathy, nephropathy, peripheral neuropathy and atherosclerosis,^4^ most of which are the micro‐ or macro‐vasculature‐related abnormalities that have been linked to a common disorders, such as vascular dysfunction, which is mainly characterized by high vascular permeability.^5^, ^6^, ^7^ It is under‐recognized that with up to 75% of young asymptomatic diabetic individuals have echocardiographic evidence of vascular dysfunction.^8^ The prevention and treatment of T2DM with vascular dysfunction is of vital significance for public health and should be further studied.

Ketogenic diet (KD) intervention is widely used as a therapeutic approach for various conditions ranging from the treatment of neurological disorders to attempts to extend lifespan.^9^ Recently, compelling evidence has suggested that KD could be attributed to blood glucose control in diabetic patients.^10^, ^11^ Typically, a KD recommends that only 5% of calories come from carbohydrates, along with 75% from fat and 20% from protein.^12^ Due to reducing the inflammatory state, a KD may have a direct beneficial effect on endothelial dysfunction and cardiovascular function.^13^, ^14^ However, the mechanisms through which the KD exerts its anti‐vascular dysfunction effects in type 2 diabetic conditions have not been fully elucidated.

Our previous study found that hepatic pescadillo1 (PES1) levels in type 2 diabetic patients and mice were significantly increased.^15^, ^16^ The increase could be inhibited by a KD.^15^ PES1, also known as pescadillo ribosomal biogenic factor 1, or NOP7 or YPH1, was first discovered in zebrafish embryos.^17^ Evidence from gastric cancer cells showed that silencing PES1 obviously decreased angiogenesis‐related gene expression.^18^ Therefore, an association was assumed between KD treatment and PES1 downregulation that modulates vascular function in T2DM mice.

In the current study, we mainly assessed the effect of a KD on diabetic vascular function and the effect of β‐hydroxybutyric acid (β‐HB) on vascular endothelial cells by downregulating PES1 expression, which may be related to the improvement in vascular hyperpermeability in vivo. Moreover, *Pes1* was knocked down in vitro and knocked out in vivo or overexpressed in vitro to explore the causal relations. Therefore, a new mechanism of PES1‐mediated vascular endothelial function influenced by KD intervention in T2MD would be unravelled. Our findings may provide a promising strategy to treat type 2 diabetic patients with vascular hyperpermeability.

## 2 METHODS

### 2.1 Mouse handling

The current study was performed with approval from the Animal Care and Use Committee of Anhui Medical University in compliance with the ethical and moral standards of laboratory animals (serial number: LLSC 20212467).

Six‐week‐old male C57BL/6J (*n* = 24) and C57BLKS/J *db/db* (*n* = 24) mice were purchased from Changzhou Cavens. All animals were housed under a 12‐h light/12‐h dark cycle and maintained within a temperature range of 20–26°C and relative humidity of 50% ± 5%, with ad libitum access to standard food (protein: 27.38%; fat: 14.50%; carbohydrate: 58.12%) and water. After 2 weeks of adaptation, all mice were randomly divided into four groups (12 mice per group), including C57BL/6J mice fed a standard diet (SD, LAD3001G, Trophic Animal Feed High‐Tech Co., Ltd, China) (C57BL/6J‐SD) or a KD (TP 201455, Trophic Animal Feed High‐Tech Co., Ltd, China) (C57BL/6J‐KD) and *db/db* mice fed a SD (*db/db*‐SD) or a KD (*db/db*‐KD). The ingredients of the KD were consistent with those described in our recent report.^15^ All mice were ad libitum accessed to water. The fresh food and water intake, body weight and fasting plasma glucose (FPG) were measured as previously described.^15^, ^16^

### 2.2 Tissue and blood sample collection

By the end of intervention, all fasted mice were euthanized to obtain tissues and blood samples. The serum was separated by centrifugation at 3000 rpm and 4°C for 15 min and stored at −80°C. The vascular tissues were rinsed with cold phosphate‐buffered saline (PBS). Small portions of vascular tissues were fixed in 4% paraformaldehyde solution for haematoxylin and eosin staining. The remaining of vascular tissues per mouse were immediately frozen in liquid nitrogen and kept at −80°C for immunoblotting. Plasma β‐HB was determined using assay kits purchased from Nanjing Jiancheng Bioengineering Institute (Jiangsu, China) in accordance with the manufacturer's instructions.

### 2.3 Cell culture and treatment

Mouse vascular endothelial cells (MVECs; cat# C166, ATCC) were cultured in cell medium supplemented with 10% FBS and 1% penicillin–streptomycin solution and maintained in an incubator with 5% CO_2_ and 95% O_2_ at 37°C. The cells were incubated in 30 mM D‐glucose medium (HG), and 30 mM D‐glucose medium plus β‐HB (HG + β‐HB). The treatment duration and optimal concentration of β‐HB were determined using the cell counting kit‐8 (CCK‐8) purchased from Nanjing Jiancheng Bioengineering Institute (Jiangsu, China) in accordance with the manufacturer's instructions, as described in our previous study.^15^

### 2.4 Western blotting

Total proteins were extracted from the abdominal aorta of mice or from MVECs using RIPA lysis buffer containing protease and phosphatase inhibitors. The abdominal aorta was crushed using an ultrasonic cell pulverizer (scientz‐IID, scientz) to obtain the supernatant (proteins). Next, the protein concentration was quantified using a BCA assay kit. A 10% SDS‐PAGE gel was used to separate the protein. Then, the protein was transferred to polyvinylidene fluoride membranes. After being blocked with 5% skim milk, the membranes were incubated with primary antibodies at 4°C overnight. The membrane was then incubated with secondary HRP‐conjugated antibody for 1 h after being washed 10 min/each for three times with TBST buffer. Blots were then developed using a chemiluminescent kit according to the manufacturer's instructions. Antibodies against the following proteins were employed: VEGF (1:1000, #ab32152, Abcam), Angiopoietin 1 (Ang‐1) (1:1000, #ab 183701, Abcam), Occludin (1:500, #NBP1‐87402, Novus), VE‐cadherin (1:1000, # sc‐9989, Santa Cruz), PES1 (1:500, #NBP2‐55211, Novus) and β‐actin (1:5000, # A5441, Sigma), respectively.

### 2.5 Vascular permeability assay in vivo

Evans blue dye was used to trace the albumin extravasation to evaluate the alteration in abdominal aorta permeability. The anesthetised mice were injected with warm Evans blue dye (2%, 45 mg/kg body weight) through the jugular vein. After the dye was circulated in the blood for 1 h, the abdominal aorta was rapidly isolated and rinsed repeatedly with phosphate buffer in the dark. Consequently, the stained abdominal aorta was photographed, dried and weighed. The dye was extracted from the aorta or blood with deionized formamide, and the absorbance at 620 nm was measured with a microplate reader for statistical analysis.^19^

### 2.6 Measurement of vascular endothelial cell permeability in vitro

Matrigel (100 μL) (the mixed base glue, 200–300 μg/mL) was used to coat the Transwell inserts and incubated at 37°C for 2 h. To allow the cells to adhere to the insert bottom, MVECs were suspended within the serum‐free medium, and then seeded in the upper insert chamber at a density of 2 × 10^5^/mL and cultured at 37°C for 1 h. Serum‐containing medium was added to the lower chamber (1 mL) and upper chamber (200 μL), respectively. MVECs were cultured in an incubator at 37°C and 5% CO_2_ for 24 h. All Transwell inserts were shifted into a new sterile 24‐well supporting plate containing 1 mL fresh serum medium, and 150 μL fluorescein isothiocyanate (FITC)‐dextran (10 μg/mL) was then added to the upper chambers and then incubated at room temperature for 25 min. The paracellular permeability was identified by the amount of FITC‐dextran diffused into the lower wells, which was detected by the fluorescence intensity of the medium using an excitation of 485 nm and an emission of 535 nm.

### 2.7 Immunofluorescence staining of cells

The expression of VEGF, PES1, VE‐cadherin and Occludin in the vascular endothelial cells was detected using immunofluorescence staining. MVECs were seeded onto 6‐well plates containing cell climbing slides for 24 h. After treatment, the cells were washed three times with PBS and fixed with 4% paraformaldehyde for 25 min. After being washed three times with PBS, the cells were supplemented with 0.5% Triton X‐100 (Ebiogo, B003, 11162115) and incubated at 37°C for 30 min. Subsequently, the 0.5% Triton X‐100 was removed, and the cells were slowly washed three times with PBS. Goat serum blocking solution (Ebiogo, B010, 10262109) was added to the cells and incubated in an incubator at 37°C for 1 h. At the end of blocking, the blocking solution was directly aspirated. The double primary antibodies were mixed according to the corresponding dilution ratio and then added into the cultured cells and incubated at 37°C for 60 min. The primary antibodies were removed, and the cells were washed slowly with PBS for three times. Alexa Fluor 488 (ab150077, Abcam) or Alexa Fluor 568 (ab175473, Abcam) conjugated secondary antibodies were added and incubated at 37°C for 30 min in the dark. The secondary antibodies were removed, and the cells were washed slowly with PBS for three times. The cell climbing slides were removed from 6‐well plates and sealed with anti‐fluorescence quenching tablets (including DAPI). A digital slice scanner (Pannoramic MIDI) was used to scan the fluorescent slices.

### 2.8 RNA interference of *Pes1*

Short interfering RNA (siRNA) was purchased from GENERAL BIOL. MVECs were seeded uniformly into 6‐well plates and allowed to grow to a density of 70%–80% for 24 h. *Pes1* siRNA was mixed with Lipofectamine 3000 for 20 min in DMEM without serum. The mixture was added into cell medium without serum and penicillin/streptomycin to transfect the MVECs for 6 h. After transfection, the medium in the 6‐well plate was replaced by fresh and complete medium with serum and penicillin/streptomycin, in which the transfected cells grew for 48 h. The cells were then harvested for running Western blotting experiments. The sequences of the *Pes1* siRNA were 5′‐ggaggaagaucggaagaaattt‐3′ (forwards) and 5′‐ uuucuuccgaucuuccucctt‐3′ (reverse).

### 2.9 Overexpression of *Pes1* in vitro

*Pes1* overexpression plasmid was obtained from GENERAL BIOL. The plasmid of *Pes1*‐flag plasmid (5 μg) and Lipofectamine 3000 (5 μL) were mixed in serum‐free medium (125 μL) for 20 min. Then, the mixture was used to transfect MVECs for 6 h. After being cultured in fresh complete medium for 24 h, the cells were divided into three groups for different treatments, including the negative control group (without *Pes1* plasmid or any reagent), *Pes1*‐plasmid group and *Pes1*‐plasmid plus β‐HB group. After being cultured for 24 h, cells were collected for Western blotting analysis.

### 2.10 Knockout of the *Pes1* gene in mice

*Pes1* knockout (KO) mice were generated in collaboration with the Nanjing Institute of Biomedicine, Nanjing University (Jiangsu, China). Genomic DNA extracted from murine tails at 4 weeks of age was used to genotype the mice by PCR. The mice were categorized into wild‐type littermate (wt/wt) and *Pes1* ^(+/−)^ (null/wt) depending on the genotypes. The primer sequences for the null mice were as follows: null sense, 5′‐ttcctcaccctcagcatttag‐3′; null antisense, 5′‐caaccaagcaaacaagaaattctc‐3′. Only male mice were opted in this study (10–12 mice per group). After 20 weeks of age, mice were sacrificed after being anaesthetized to obtain the abdominal aorta for Western blotting.

### 2.11 Co‐immunoprecipitation and ubiquitination assays

The binding of PES1 to VE‐cadherin was measured by co‐immunoprecipitation (co‐IP) assay. PES1‐mediated VE‐cadherin ubiquitination was analysed after treatment with *Pes1*‐siRNA or the *Pes1* overexpression plasmid. The detailed methods were referred to our previous reports.^15^, ^16^

### 2.12 Statistical analyses

Statistical analyses were conducted using SPSS 22.0. All results are presented as the mean ± SEM. Student's *t*‐test was used for the analysis of two groups, and anova followed by the Student–Newman–Keuls *q*‐test was used to compare multiple groups. Statistical significance was defined as *p* < 0.05. Graphs were prepared using GraphPad Prism, version 8 (GraphPad Software).

## 3 RESULTS

### 3.1 KD intervention sharply decreased the hyperglycaemia in diabetic mice

**FIGURE 1:**
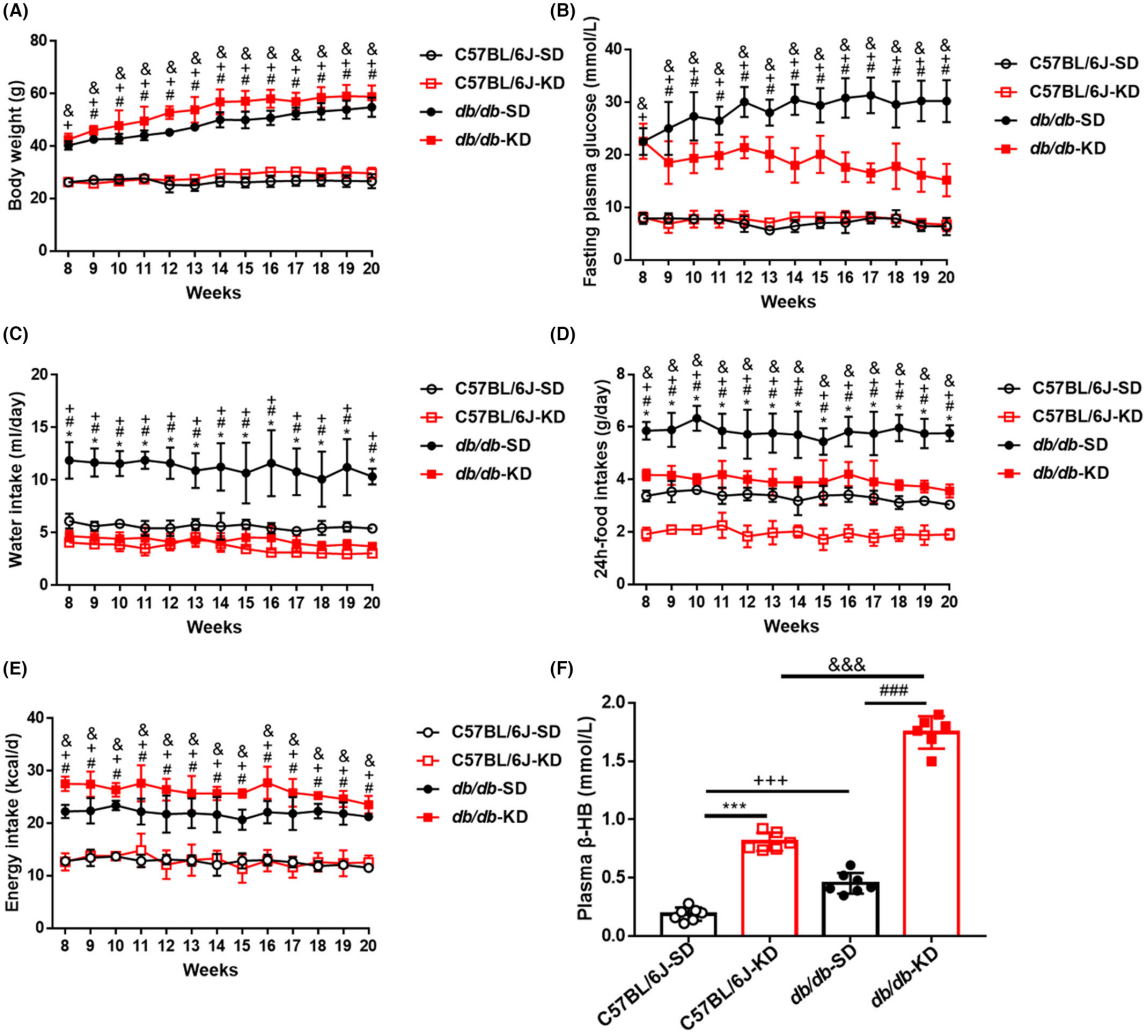
KD intervention significantly decreased the hyperglycaemia and energy intakes in diabetic mice. (A) Exhibited are the changes of body weights exerted by the different food feedings in normal and diabetic mice. (B) Unveiled are the variations of fasting plasma glucose in different groups throughout 12 weeks. (C) Shown are the food intakes in different groups throughout 12 weeks of feeding. (D) Displayed are the water intakes in different groups throughout 12 weeks. (E) Demonstrated are the energy intakes in different groups throughout 12 weeks. (F) Shown are the levels of plasma β‐hydroxybutyric acid (β‐HB) in different groups by SD or KD intervention. Data are represented as mean ± SEM, each assay was performed independently three times (*n* = 12 per group). KD (ketogenic diet), SD (standard diet). *p < 0.05 C57BL/6J‐KD versus C57BL/6J‐SD, ^#^ *p* < 0.05 *db/db*‐KD versus *db/db*‐SD, ^+^ *p* < 0.05 C57BL/J‐SD versus *db/db*‐SD, ^&^ *p* < 0.05 C57BL/6J‐KD versus *db/db*‐KD (anova, Student–Newman–Keuls *q*‐test).

After 12 weeks of intervention, murine body weights in the C57BL/6J‐KD group exhibited no significant difference from those in the C57BL/6J‐SD group. The *db/db*‐SD group did clearly show higher body weights relative to the C57BL/6J‐SD group, nonetheless, KD intervention could slightly enhanced the body weights of *db/db* mice (Figure 1A). The FPG levels were markedly enhanced in diabetic mice by SD feeding compared to normal mice with SD feeding (Figure 1B). However, the levels were significantly reduced in diabetic mice by the KD intervention. Furthermore, the mice in the C57BL/6J‐KD group displayed no significant difference in FPG from those in the C57BL/6J‐SD group (Figure 1B). For both normal and diabetic mice, the food intake of the SD mice was significantly higher than that of the KD mice, parallel to the consumption of water (Figure 1C,D). Moreover, the SD food intakes and water consumption in diabetic mice was markedly higher than that in normal mice (Figure 1C,D). In terms of energy intake, the SD‐fed *db/db* mice showed a dramatic increase, which was further enlarged in KD‐fed *db/db* mice (Figure 1E). However, the difference was not observed in SD‐ and KD‐fed C57BL/6J mice. In addition, although the plasma β‐HB levels were increased in SD‐fed diabetic mice, they were drastically elevated by KD feeding in both healthy and diabetic mice (Figure 1F).

### 3.2 KD improved vascular hyperpermeability and reduced vascular stiffness and leakage in type 2 diabetic mice

**FIGURE 2:**
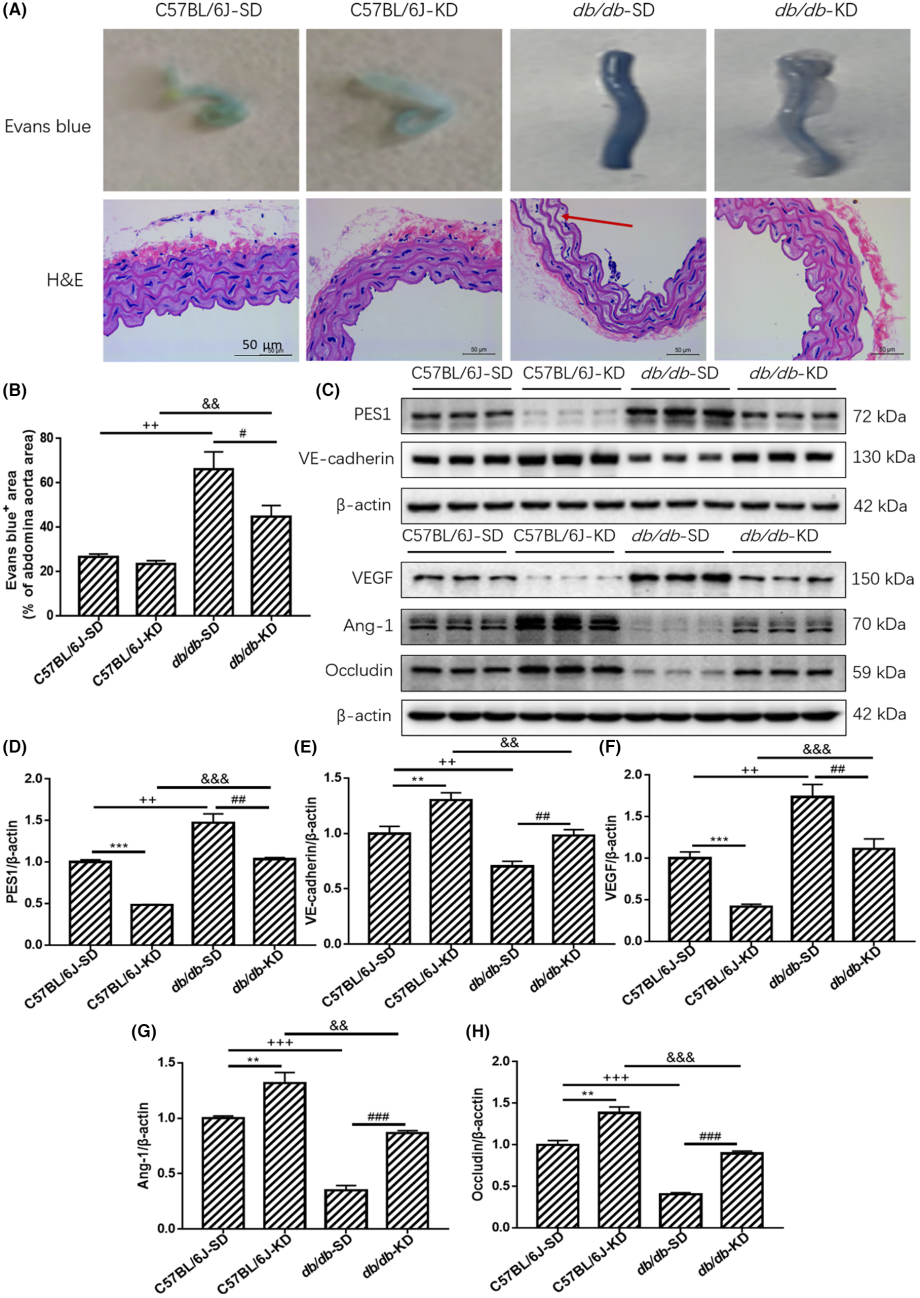
KD improved vascular hyperpermeability and reduced vascular stiffness and leakage in type 2 diabetic mice. (A, B) The Evans blue injection and haematoxylin and eosin staining of abdominal aorta were performed for different groups, original magnification, ×10 (haematoxylin and eosin staining). Scale bar, 50 μm (haematoxylin and eosin staining). (C–H) The protein levels of vascular PES1, VEGF, VE‐cadherin, Ang‐1 and Occludin were detected by Immunoblotting. Data are represented as mean ± SEM, each assay was performed independently three times (*n* = 12 per group). KD (ketogenic diet), SD (standard diet). **p < 0.01 C57BL/6J‐KD versus C57BL/6J‐SD, ***p < 0.001 C57BL/6J‐KD versus C57BL/6J‐SD, ^#^ *p* < 0.05 *db/db*‐KD versus *db/db*‐SD, ^##^ *p* < 0.01 *db/db*‐KD versus *db/db*‐SD, ^###^ *p* < 0.001 *db/db*‐KD versus *db/db*‐SD, ^+^ *p* < 0.05 C57BL/J‐SD versus *db/db*‐SD, ^++^ *p* < 0.01 C57BL/J‐SD versus *db/db*‐SD, ^+++^ *p* < 0.001 C57BL/J‐SD versus *db/db*‐SD, ^&&^ *p* < 0.01 C57BL/6J‐KD versus *db/db*‐KD, ^&&&^ *p* < 0.001 C57BL/6J‐KD versus *db/db*‐KD (anova, Student–Newman–Keuls *q*‐test).

To examine the leakage of blood vessels in normal and diabetic mice, Evans blue injection was used to test abdominal aorta permeability. As shown in Figure 2A,B, the angiography demonstrated that the vascular permeability of the abdominal aorta in the SD‐fed *db/db* mice was elevated, which was markedly reversed by KD treatment (Figure 2A,B). To observe the pathological alteration in diabetic abdominal aorta caused by KD intervention, haematoxylin and eosin staining was performed. The results revealed that the abdominal aorta wall of diabetic mice was thicker than that of normal mice, while KD treatment significantly improved the pathogenic thickness and morphological changes (Figure 2A). These improvements were not obviously observed in SD‐ and KD‐fed C57BL/6J mice. Additionally, the immunoblots revealed that SD‐fed diabetic mice showed dramatic increases in the protein levels of PES1 and VEGF, which were significantly suppressed by KD intervention (Figure 2C–H). In contrast, the protein levels of VE‐cadherin, Ang‐1 and Occludin were markedly lowered in SD‐fed diabetic mice, while these reductions were sharply improved by KD intervention. Therefore, our current findings suggested that vascular hyperpermeability caused by diabetes in mice could be effectively ameliorated by KD intervention.

### 3.3 β‐HB treatment reduced vascular endothelial paracellular permeability in vitro

**FIGURE 3:**
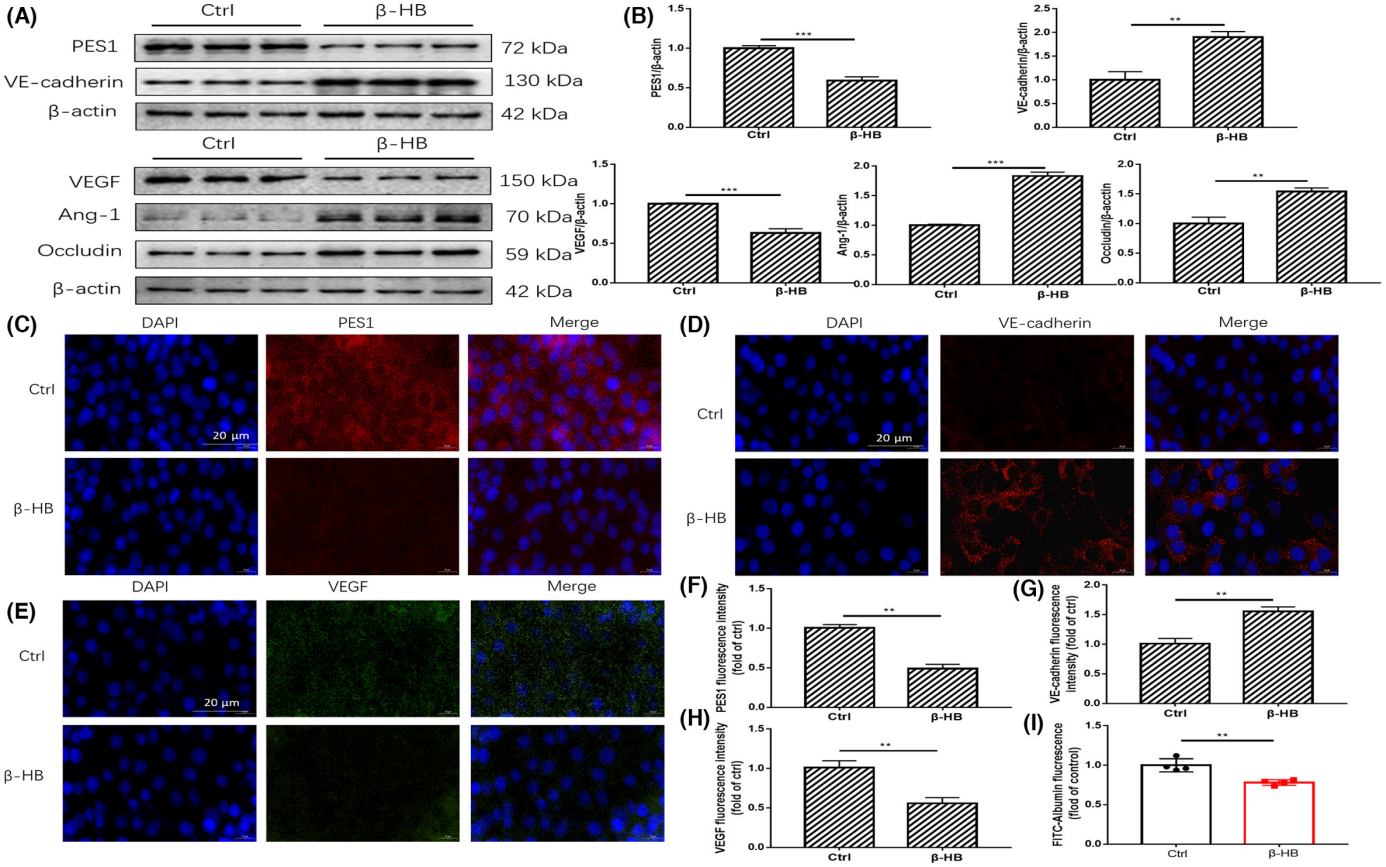
β‐HB treatment reduced vascular endothelial paracellular permeability in vitro. (A, B) The protein levels of PES1, VEGF, VE‐cadherin, Ang‐1 and Occludin in MVECs were detected by immunoblotting after 2 mM β‐HB treatment for 24 h. (C–H) Displayed are immunofluorescence images of β‐HB‐treated MVECs for VE‐cadherin, VEGF and PES1 expression and localizations, scale bar represents 20 μm. The nuclei were stained with DAPI. (I) Exhibited is the paracellular permeability in the cultured MVECs under different treatments. Ctrl (Control), β‐HB (β‐hydroxybutyric acid). Data were represented as mean ± SEM, each experiment was performed independently three times. **p < 0.01, ***p < 0.001 compared with control (Student's *t*‐test).

β‐HB was used to simulate the effect of a KD on MVECs in vitro. The optimal treatment time (24 h) and concentration (2 mM) of β‐HB were determined by the CCK‐8 assay (Figure S1). Western blotting demonstrated that the protein expression of PES1 and VEGF in the β‐HB‐treated group was lower than that in the control group (Figure 3A,B). Whereas, the protein levels of VE‐cadherin, Ang‐1 and Occludin in MVECs were markedly increased after β‐HB treatment (Figure 3A,B). Consistent with the above‐mentioned data, the immunofluorescence staining results showed that β‐HB suppressed PES1 and VEGF, while enhanced VE‐cadherin protein expression in MVECs (Figure 3C–H). Concomitantly, changes in vascular paracellular permeability in β‐HB‐treated MVECs were detected using the Transwell assay. Compared with the control group, β‐HB treatment significantly decreased the leakage of FITC‐dextran (Figure 3I), suggesting an obvious amelioration of vascular endothelial paracellular permeability in MVECs.

### 3.4 In vitro knockdown of *Pes1* lowered the paracellular permeability in MVECs

**FIGURE 4:**
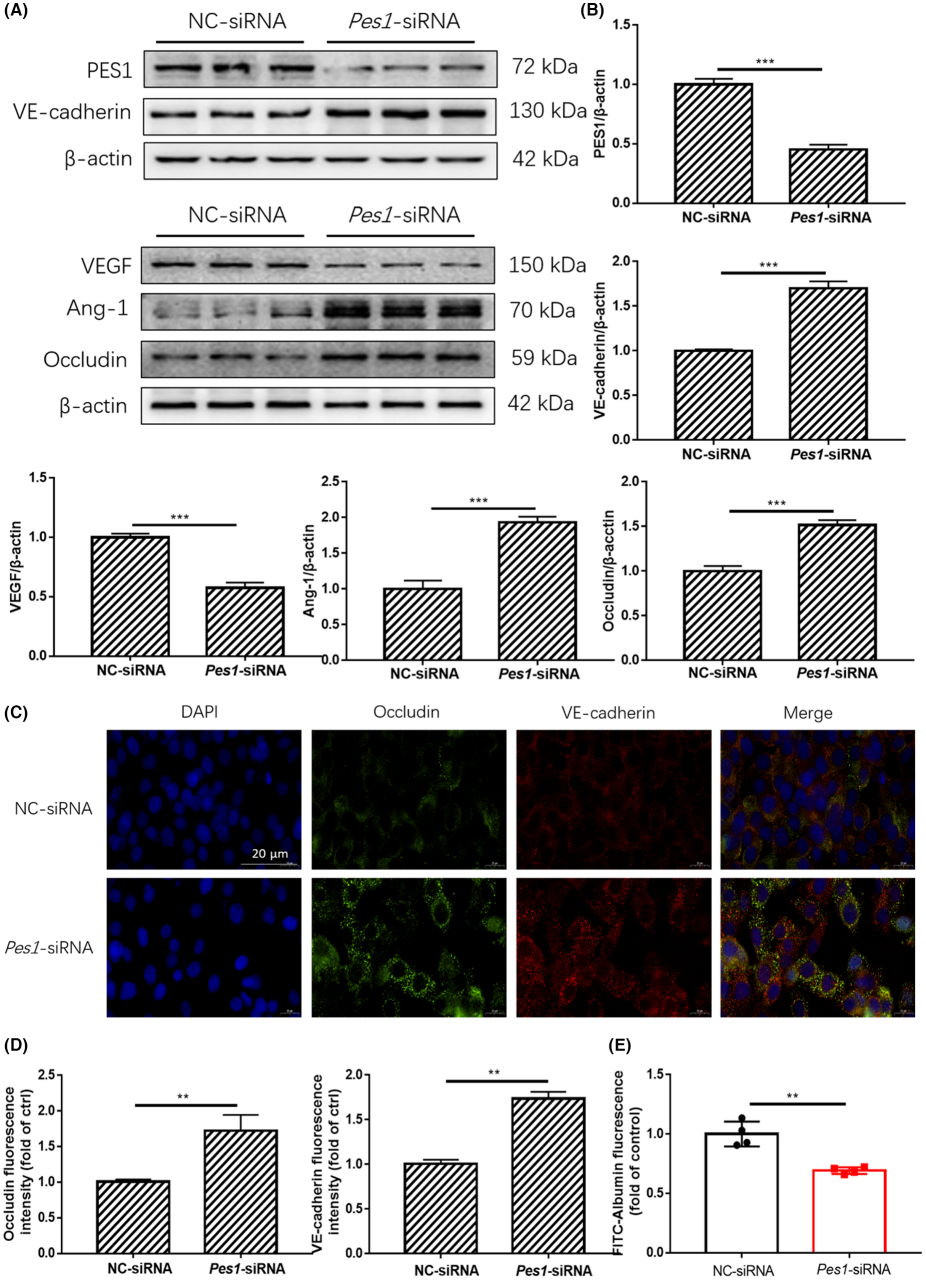
In vitro knockdown of *Pes1* lowered the paracellular permeability of MVECs. (A, B) The protein levels of PES1, VEGF, VE‐cadherin, Ang‐1 and Occludin in MVECs were detected by immunoblotting after *Pes1*‐siRNA treatment. (C, D) Shown are immunofluorescence images of *Pes1*‐siRNA‐treated MVECs for Occludin and VE‐cadherin expression and localizations, scale bar represents 20 μm. The nuclei were stained with DAPI. (E) Exhibited is the paracellular permeability in the cultured MVECs in different groups. Data were represented as mean ± SEM, each experiment was performed independently three times. **p < 0.01, ***p < 0.001 compared with control (Student's *t*‐test).

To determine whether PES1 can play an obligatory role in regulating vascular permeability in MVECs, we used siRNA to knock down *Pes1* in MVECs. After *Pes1* was silenced by siRNA, Western blotting showed that the expression of PES1 and VEGF was significantly decreased (Figure 4A,B). Conversely, the levels of VE‐cadherin, Ang‐1 and Occludin were enhanced. Coherent with the aforementioned data, the immunofluorescence staining results showed that knockdown of *Pes1* increased VE‐cadherin and Occludin expression in MVECs (Figure 4C,D). Simultaneously, vascular endothelial paracellular permeability after *Pes1*‐siRNA treatment was significantly lowered than that after NC‐siRNA treatment (Figure 4E). Taken together, these results indicated that PES1 may exert an important role in upregulating vascular endothelial paracellular permeability.

### 3.5 *Pes1* knockout in mice decreased vascular permeability

**FIGURE 5:**
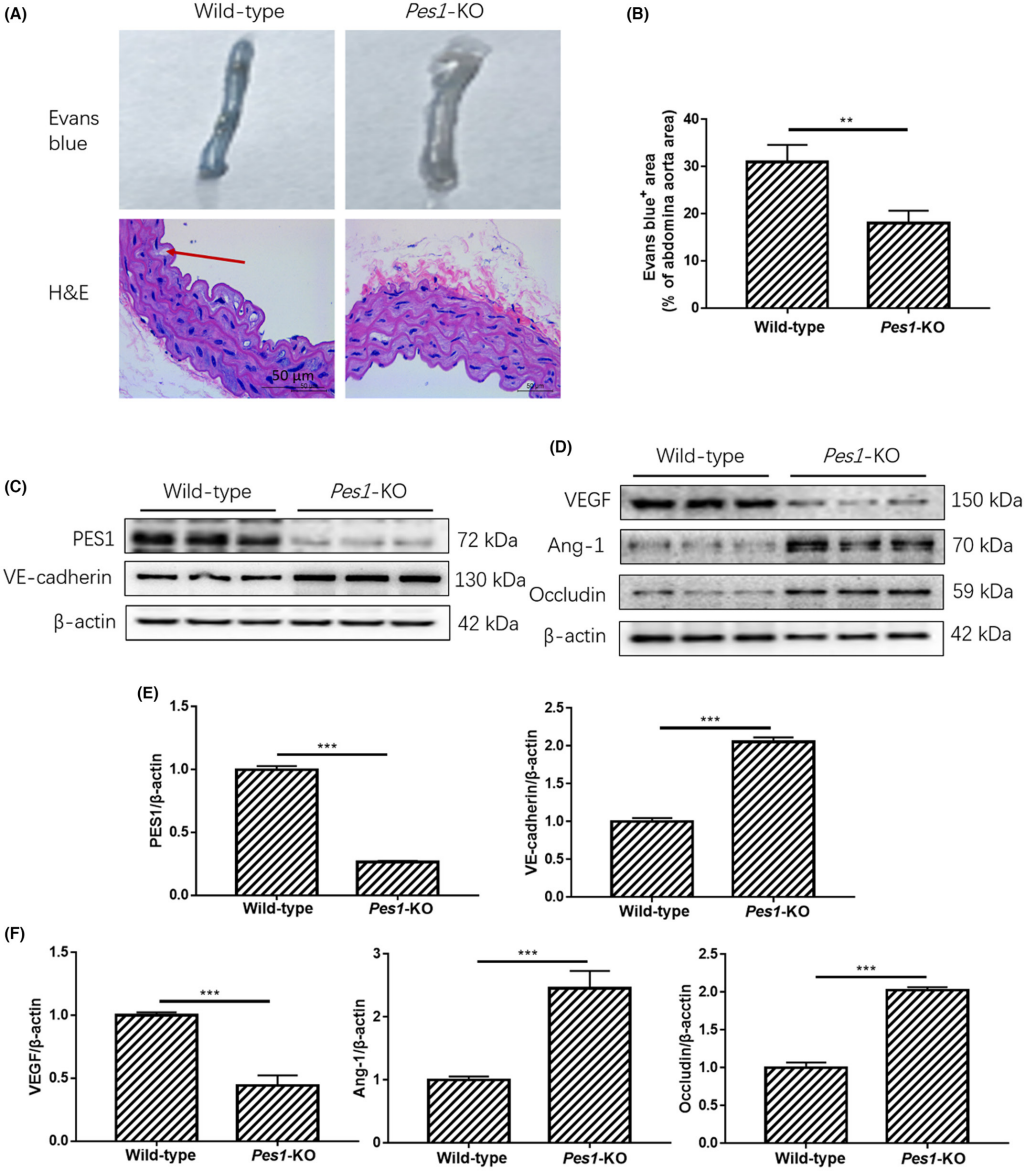
*Pes1* knockout in mice decreased vascular permeability. (A, B) The Evans blue injection and haematoxylin and eosin staining of abdominal aorta were conducted in different groups, original magnification, ×10 (haematoxylin and eosin staining). Scale bar, 50 μm (haematoxylin and eosin staining). (C–F) The protein levels of vascular PES1, VEGF, VE‐cadherin, Ang‐1 and Occludin were measured by Immunoblotting. Data were represented as mean ± SEM, each assay was performed independently three times. **p < 0.01, ***p < 0.001 compared with control (Student's *t*‐test).

Based on the above results, PES1 may be a vital booster of hyperpermeability in diabetic mice or of paracellular permeability in cultured MVECs under HG conditions. To further verify the role of PES1 in regulating vascular permeability in vivo, *Pes1*‐KO mice were employed. After 20 weeks of feeding, Evans blue injection demonstrated that the pathological changes and vascular permeability in the *Pes1*‐KO mice were significantly improved compared with those in their wild‐type littermate (Figure 5A,B). Haematoxylin and eosin staining showed that the vascular structure of the aorta in both groups was clear and that the cells were closely arranged. However, the vascular smooth muscle and elastic membrane space in *Pes1*‐KO mice were tighter than those in their wild‐type littermate (Figure 5A). In addition, immunoblots demonstrated that the protein expression of VEGF and PES1 was sharply decreased in the abdominal aorta of the *Pes1*‐KO mice (Figure 5C,E), but the protein expression of VE‐cadherin, Ang‐1 and Occludin was significantly increased (Figure 5D–F).

### 3.6 β‐HB treatment eliminated the elevation of paracellular permeability by in vitro supplementation of *Pes1*

**FIGURE 6:**
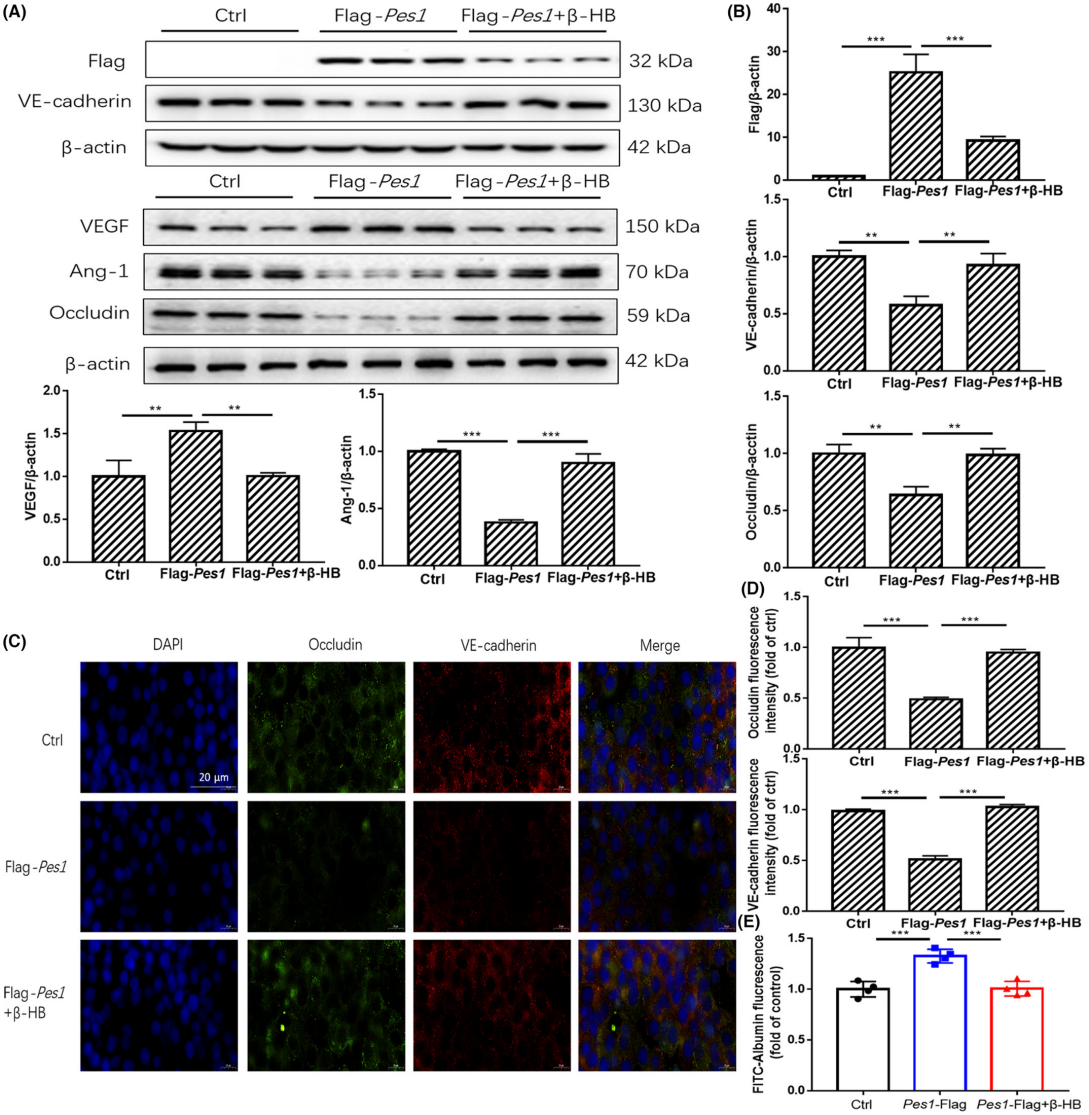
β‐HB treatment impaired the increment of paracellular permeability by in vitro supplementation of *Pes1*. (A, B) The protein levels of PES1, VEGF, VE‐cadherin, Ang‐1 and Occludin in MVECs were detected by immunoblotting after Flag‐*Pes1* plus β‐HB treatment. (C, D) Shown are immunofluorescence images of Flag‐*Pes1* plus β‐HB‐treated MVECs for Occludin and VE‐cadherin expression and localizations, scale bar represents 20 μm. The nuclei were stained with DAPI. (E) Exhibited is the paracellular permeability in the cultured MVECs in different groups. Data were represented as mean ± SEM, each experiment was performed independently three times. **p < 0.01, ***p < 0.001 compared with control (anova, Student–Newman–Keuls *q*‐test).

The overexpression of *Pes1* in vitro was performed to further confirm the key role of PES1 in vascular permeability in MVECs. Our current results showed that the protein expression of the VEGF and PES1 was significantly increased and that VE‐cadherin, Ang‐1 and Occludin were notably decreased by *Pes1* overexpression treatment, but these changes were sharply reversed by β‐HB treatment (Figure 6A,B). Similar results were also observed by immunofluorescence staining. The observation showed that overexpression of *Pes1* significantly decreased the protein expression of VE‐cadherin and Occludin, while β‐HB treatment eliminated this reduction (Figure 6C,D). Transwell assays revealed that β‐HB treatment impaired the increase in FITC‐dextran leakage after *Pes1* overexpression (Figure 6E). These results indicated that β‐HB reduced paracellular permeability by downregulating the PES1 expression.

### 3.7 β‐HB treatment in MVECs decreased PES1‐facilitated VE‐cadherin ubiquitination

**FIGURE 7:**
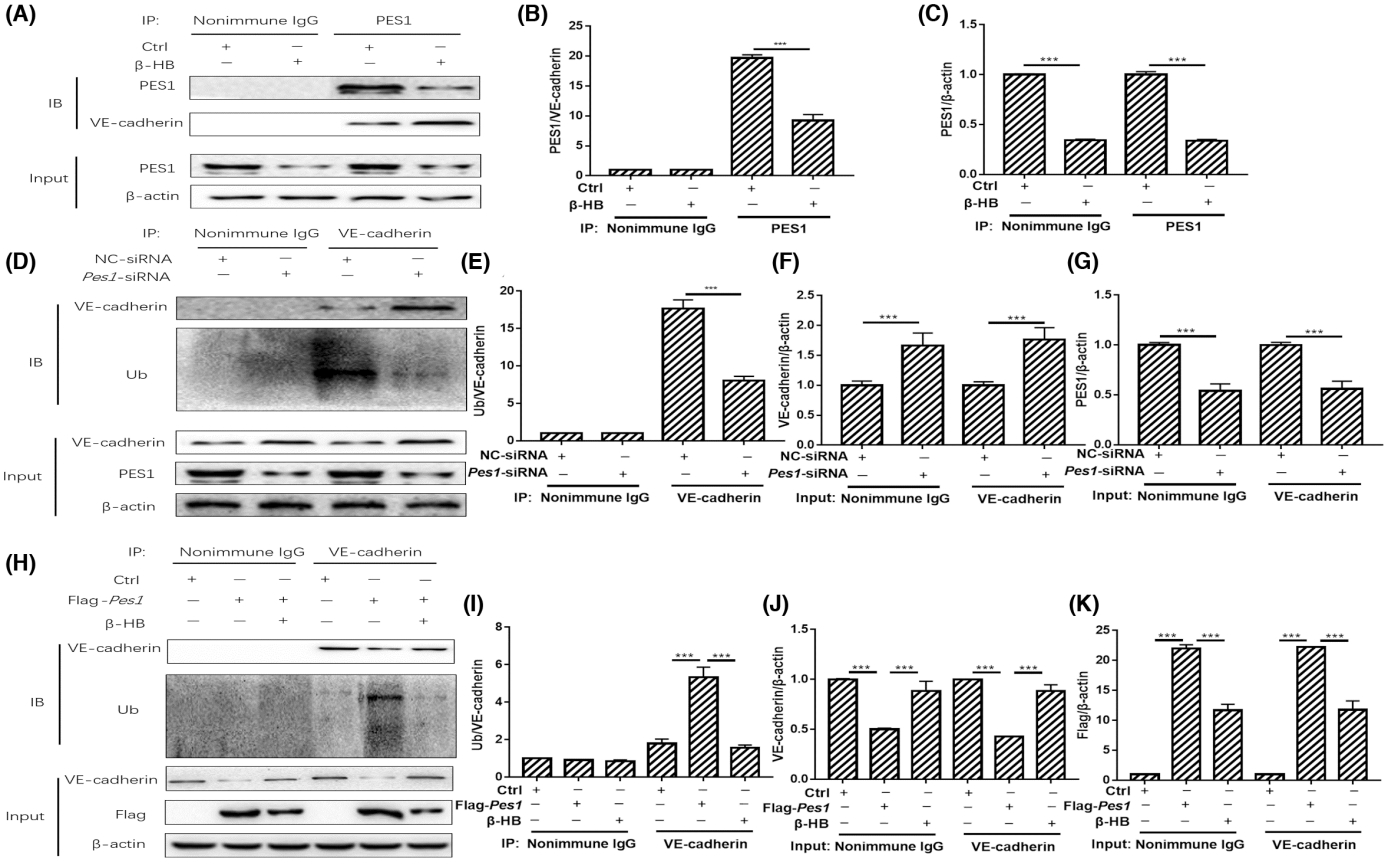
β‐HB treatments decreased PES1‐facilitated VE‐cadherin ubiquitination in MVECs. (A–C) β‐HB increased the interaction between PES1 and VE‐cadherin. (D–G) Knockdown of *Pes1* in cultured cells extenuated the ubiquitination of VE‐cadherin. (H–K) Overexpression of *Pes1* in cultured cells promoted the ubiquitination of VE‐cadherin, which was curbed by β‐HB treatment. Each experiment was performed independently three times. ***p < 0.001 compared with control (anova, Student–Newman–Keuls *q*‐test).

To explore whether PES1 directly interacts with VE‐cadherin, co‐IP assays were conducted on cultured MVECs. We discovered that far more VE‐cadherin protein associated with PES1 in β‐HB‐treated MVECs than in control cells (Figure 7A–C). To further investigate PES1‐mediated VE‐cadherin stability, we assessed whether PES1 affects the ubiquitination of VE‐cadherin. As exhibited in Figure 7B, the ubiquitination of VE‐cadherin was strikingly inhibited by *Pes1* knockdown in cultured cells (Figure 7D–G). In contrast, overexpression of *Pes1* in cultured MVECs elevated the ubiquitination of VE‐cadherin, which was reversed by β‐HB treatment (Figure 7H–K). Therefore, β‐HB treatments of MVECs may substantially lower PES1‐facilitated VE‐cadherin ubiquitination.

## 4 DISCUSSION

Our current study demonstrated that KD intervention markedly ameliorated vascular hyperpermeability via inhibiting the vascular PES1 in diabetic mice, consistent with the β‐HB treatment in vitro. Moreover, the levels of VEGF and PES1 were obviously reduced, but VE‐cadherin, Ang‐1 and Occludin were significantly increased in *Pes1*‐siRNA‐treated MVECs and in the abdominal aorta of *Pes1*‐KO mice, in contrast to the *Pes1* overexpression in cultured MVECs. Mechanistically, our current data indicated that PES1 could interact with VE‐cadherin and thereby facilitate the ubiquitination of VE‐cadherin, which was curbed by β‐HB treatment. Collectively, these results suggested that KD may ameliorate vascular hyperpermeability in diabetic mice by downregulating vascular PES1.

Published clinical trial and animal model studies have implied that a KD was effective in improving the metabolic parameters of T2DM, including body weight, glycaemia, plasma lipid profiles, insulin resistance and HbA1c (%).^20^, ^21^, ^22^ In this study, FPG levels in *ob/ob* mice were significantly improved by KD intervention. However, the body weights in KD‐fed *db/db* mice even showed a slight increase compared with the SD‐fed diabetic mice, which was consistent with previous publications.^23^, ^24^ The reason may be that the *db/db* mice used in this study are a spontaneous type 2 diabetic model caused by a defect in the leptin receptor gene located on chromosome 4,^25^ thus stimulating appetite, as shown in our data for energy intake. Emerging evidence from an experimental study reported that a KD substantially dropped the vascular permeability in a mouse glioma model,^26^ which exactly resembles our current study.

We discovered that KD intervention markedly increased the levels of VE‐cadherin, Ang‐1 and Occludin, but extenuated the expression of VEGF in both normal and diabetic mice. VE‐cadherin is an endothelial cell‐specific adherent junction protein that is essential for the vascular endothelial permeability and leukocyte entry into tissues.^27^ Ang‐1 has a role in vascular maturation, stabilizing endothelial interactions with supporting cells and limiting vascular permeability.^28^ Occludin, the most representative tight junction protein, could maintain the integrity and physiological permeability of the vascular endothelium.^29^ VEGF increases vascular permeability, promotes cell migration and has mitotic and anti‐apoptotic effects on endothelial cells.^30^ All of these changes observed in our current study suggested that KD intervention may improve the vascular endothelial function through regulating those factors, consistent with the results from β‐HB‐treated cells.

β‐HB, as the predominant component of ketone bodies, can inhibit vascular hyperpermeability,^31^ as shown in our current data from KD‐fed *db/db* mice and β‐HB‐treated MVECs. The plasma levels of β‐HB in KD‐fed C57BL/6J and *db/db* mice were sharply higher than those in SD‐fed both of mice, indicating that the KD elicited ketogenesis in vivo. Of note, the levels of vascular PES1 in KD‐fed diabetic mice and β‐HB‐treated MVECs were significantly suppressed. Our recent study found that hepatic PES1 levels in either type 2 diabetic or obese patients and in either type 2 diabetic or obese mice were significantly increased.^15^, ^16^, ^32^ In addition, previous report showed that PES1 could regulate angiogenesis‐related gene expression in gastric and ovarian cancer.^18^, ^33^ Moreover, our current in vivo and in vitro data firmly support the causal relation between PES1 expression and the downregulation of VE‐cadherin, Ang‐1 and Occludin, thereby linking the role of PES1 to modulating vascular permeability and vascular endothelial function.

More interestingly, we observed that β‐HB substantially augmented the interaction between PES1 and VE‐cadherin and that silencing endogenous *Pes1* by siRNA significantly inhibited the ubiquitination of VE‐cadherin, leading to the increased protein stability and decreased vascular permeability. Conversely, supplementation of exogenous *Pes1* in MVECs elevated the ubiquitination of VE‐cadherin, which was however relieved by β‐HB treatment. Therefore, mechanistically, β‐HB may improve vascular permeability by downregulating PES1‐facilitated ubiquitination of VE‐cadherin.

Here, we showed the essential role of KD intervention in diabetic mice by decreasing endothelial hyperpermeability via inhibiting vascular PES1. By targeting PES1, a clinical drug may be developed to lower vascular hyperpermeability under diabetic condition. Although the focus of our current study was on PES1 expression in the abdominal aorta, and our current results do not include the involvement of other tissues or factors responsible for vascular endothelial hyperpermeability, it cannot be denied that the KD is a promising candidate for novel therapeutic strategies.

In summary, our present results suggest that a KD may improve vascular hyperpermeability in type 2 diabetic mice by downregulating PES1. These findings may indicate the potential of targeting vascular PES1 to reduce the risk of diabetic vascular hyperpermeability.

## AUTHOR CONTRIBUTIONS

**Song Wang:** Conceptualization (equal); data curation (equal); formal analysis (equal); investigation (equal). **Jielin Zhou:** Formal analysis (lead); software (lead); supervision (lead); writing – original draft (lead); writing – review and editing (lead). **Jing Lu:** Software (equal); validation (equal); visualization (equal). **Yan Lin:** Investigation (equal); project administration (equal); software (equal). **Shuaishuai Liu:** Investigation (equal); software (equal); supervision (equal); visualization (equal). **Keyang Chen:** Conceptualization (equal); data curation (equal); funding acquisition (lead).

## FUNDING INFORMATION

This work is supported by the National Natural Science Foundation of China (NSFC, 81570786 to Keyang Chen).

## CONFLICT OF INTEREST STATEMENT

The authors declare no competing interests.

## Supporting information


Figure S1.

## Data Availability

The data that support the findings of this study are available from the corresponding author upon reasonable request.
