# Supplementary material for: A ketogenic diet improves vascular hyperpermeability in type 2 diabetic mice by downregulating vascular pescadillo1 expression
Source: J Cell Mol Med. 2023 Apr 15;27(10):1410–22. doi: 10.1111/jcmm.17744 (PMC10183701; doi:10.1111/jcmm.17744)
Supplement: Supplementary file 1 — Figure S1. [file JCMM-27-1410-s001.docx]

**SUPPLEMENTARY** **FIGURE** **1**
